# Tongluo Yishen Decoction Ameliorates Renal Fibrosis *via* NLRP3-Mediated Pyroptosis *In Vivo* and *In Vitro*

**DOI:** 10.3389/fphar.2022.936853

**Published:** 2022-07-06

**Authors:** Qi Jia, Xiaoyu Zhang, Gaimei Hao, Yun Zhao, Scott Lowe, Lin Han, Jianguo Qin

**Affiliations:** ^1^ Department of Nephropathy, Dongfang Hospital, Beijing University of Chinese Medicine, Beijing, China; ^2^ Institute of Basic Theory for Chinese Medicine, China Academy of Chinese Medical Sciences, Beijing, China; ^3^ Kansas City University of Medicine and Biosciences, College of Osteopathic Medicine, Kansas City, MO, United States; ^4^ School of Basic Medicine, Beijing University of Chinese Medicine, Beijing, China

**Keywords:** Tongluo Yishen decoction, obstruction-induced renal fibrosis, Traditional Chinese Medicine, NRK-52E, pyroptosis

## Abstract

**Purpose:** In this study, we investigated the mechanism of Tongluo Yishen (TLYS) decoction in more detail, from the perspective of pyroptosis in the unilateral ureteral ligation (UUO) model and the hypoxia-induced renal tubular epithelial (NRK-52E) cell.

**Method:** The UUO model was used, and after 14 days of TLYS intervention, rats were tested for blood creatinine and urea nitrogen, HE staining was used to observe the pathological changes in the kidney, Masson staining was used to assess the degree of interstitial fibrosis, western blot was used to detect the changes of α-smooth muscle actin (α-SMA) protein expression level, immunohistochemistry and western blot detected the changes in protein expression levels of NOD-like receptor protein 3 inflammasome (NLRP3), gasdermin D (GSDMD), cysteinyl aspartate specific proteinase (caspase-1), interleukin 18 (IL-18) and interleukin 1β (I L-1β). A hypoxia model was created using NRK-52E cell, and after different concentrations of TLYS decoction intervention, the changes in the expression levels of pyroptosis were used with immunofluorescence and western blot methods.

**Results:** TLYS decoction improved renal function, delayed the advancement of renal interstitial fibrosis, and inhibited pyroptosis in UUO rats. Furthermore, we observed that TLYS can mitigate hypoxia-induced NRK-52E cell damage via the suppression of the NLRP3-mediated pyroptosis.

**Conclusion:** TLYS decoction exert renoprotective effects by inhibiting NLRP3-mediated pyroptosis.

## Introduction

Renal interstitial fibrosis (RIF) is a critical event in the progression of chronic kidney disease (CKD) to end-stage renal disease (ESRD) (Humphreys, 2018). Unilateral ureteral ligation (UUO) is a well-established animal model of RIF, the major pathophysiological changes involved necrosis, inflammation, activation of a variety of macrophages, cytokine release, and a large accumulation of extracellular matrix (ECM) (Chevalier et al., 2009). During the early stages of acute unilateral obstruction in this mouse model, tubular cells are immediately and dramatically injured (Klahr and Morrissey, 2002). Cell death play a dominant role in the early stages of pathological progression of acute obstructive nephropathy, according to growing evidence (Hosseinian et al., 2017; Zhang B. et al., 2021). Inflammation is a key link in the development of RIF, existing research evidence indicates that epithelial-mesenchymal transition (EMT) and fibroblast activation caused by overexpression of inflammatory factors and inflammatory cell infiltration have important pathological significance in RIF in acute or chronic kidney injury (Meng et al., 2014; Tang et al., 2019). The mobilization and infiltration of macrophages and neutrophils, as well as the subsequent release of cytokines such as interleukin-1β (IL-1β) and IL-18, all contribute significantly to the progression of fibrosis.

Pyroptosis is a newly identified type of programmed inflammatory cell death characterized by excessive cell death and inflammation that can be triggered by canonical caspase-1 inflammasomes or non-canonical caspase-4-, caspase-5-, and caspase-11-mediated pathways (Broz et al., 2020; Kesavardhana et al., 2020). When pyroptosis occurs via canonical signaling, caspase-1 is converted into its active forms (p20 and p10 subunits) by inflammasomes (NLRP3, AIM2, etc.) and then activates proinflammatory cytokines interleukin IL-18 and IL-1β to mature IL-18 and IL-1β (Yu et al., 2021). These have potent pro-inflammatory properties and promote cell vasodilation and extravasation, as well as amplifying the local and systemic inflammatory response (Swanson et al., 2019).

The traditional Chinese medicine Tongluo Yishen (TLYS) decoction has been widely used to treat CKD for decades. TLYS is a compound preparation composed of Salvia, Radix et Rhizoma, Safflower, and Cortex. The components in these herbs could be used to treat kidney disease. Several components of TLYS have been shown in numerous studies to have anti-inflammatory and antioxidant properties (Zhang et al., 2014; Xu et al., 2017; Ye T. et al., 2020; Tang et al., 2020). Our previous research found that TLYS alleviated renal pathological damage by improving oxidative stress in the kidneys of UUO rats (Jia et al., 2021). However, the mechanism of whether TLYS plays a role in pyroptosis remains unclear. Based on previous research, we hypothesized that TLYS might protect renal functions against renal fibrosis via NLRP3 inflammasome-induced pyroptosis. This hypothesis was tested in hypoxic cultures of UUO rats and NRK-52E cells.

## Materials and Methods

### Animal Model and Experimental Design

After fasting without water for 12 h prior to surgery, 2 percent sodium pentobarbital was administered intraperitoneally at 40 mg/kg according to the rat’s body weight for anesthesia, the right side of the rat’s back was selected for skin preparation and disinfection, an incision was made at 1 cm next to the right rib-spine angle, parallel to the spine, the incision length was about 0.5–0.7 cm, the skin and muscle layer were incised. The UUO group was divided into three groups: the model group, the TLYS group, and the valsartan group, with nine animals in each group receiving gavage administration. The valsartan group received 0.84 mg/100 (g·d) of valsartan. TLYS was given 0.8 g/100 (g·d) to the TLYS group. The treatment period lasted 14 days. The sham-operated and model groups were given 1 ml/100 (g·d) of distilled water daily.

### Preparation of Tongluo Yishen Decoction

TLYS decoction contains 25 g Salvia, 15 g Radix Bupleurum, 10 g Safflower, and 10 g Chrysanthemum. The herbal medicine was soaked for 60 min in 600 ml of water (10 times the mass of the herbal medicine), boiled, and then decocted for 40 min twice, combined and blended to remove the dregs, concentrated to contain 1 g of raw herbs per ml, and stored at 4°C. The herbs are used *in vivo* experiments. Place the sterilized TLYS herbs in the freeze-drying mechanism to create a freeze-dried powder. When ready to use, weigh 1 g lyophilized powder with a balance, add 1 ml ultrapure water for redissolution, mix evenly on the shaking table, transfer to a high-temperature resistant container, disinfect and sterilize it under high temperature and high pressure, and store it at 4°C for standby, current use, and distribution. The freeze-dried powder is used *in vitro* experiments.

### Measurement of Serum Creatinine (Scr) and Blood Urea Nitrogen (BUN)

Scr and BUN levels were determined using a creatinine assay kit (C011-1, Nanjing Jiancheng Bioengineering Institute, Nanjing, China) and a BUN assay kit (C013-1, Nanjing Jiancheng Bioengineering Institute, Nanjing, China) according to the manufacturer’s instructions.

### Histology and Immunohistochemistry

Histological Examination: Six kidneys from each group were immediately fixed with 10% formalin, dehydrated, embedded in paraffin, and sectioned to a thickness of 5 µm. These sections were then stained with hematoxylin and eosin (H&E) and Masson’s trichrome. Immunohistochemistry (IHC) Staining: Five-micron thick paraffin-embedded kidney sections were deparaffinized, followed by antigen retrieval in ethylenediaminetetraacetic acid (1 mM). The samples were blocked with 0.3% H2O2 in methanol and 5% BSA. Kidney sections were incubated with NLRP3 (1:200, ab263899, Abcam, United States), GSDMD (1:200, ab255603, Abcam, United States), IL-18 (1:200, 60070-1-Ig, Proteintech, United States) and IL-1β (1:200, 16806-1-AP, Proteintech, United States) primary antibodies overnight at 4°C, followed by horseradish peroxidase (HRP)-conjugated secondary antibodies (PV9001, Beijing Zhongshan Jinqiao Biotechnology Co., Ltd., Beijing). The reaction was visualized with DAB staining using a Leica Aperio Versa 8 system (Leica, Wetzlar, Germany). The cumulative optical density of the area of interest analysis was calculated using ImageJ software.

### Cell Culture and Treatment

The rat renal tubular epithelial cells (RTEC, NRK-52E) were obtained from the Chinese Academy of Sciences’ Shanghai cell bank (batch No.:20161104). NRK-52E was cultured in a 37°C, 5% CO_2_ normal incubator with 10% fetal bovine serum (Gibco, United States) and 1% double antibody (100 U/mL penicillin, 100 G/ml streptomycin, Gibco, United States), 89 percent DMEM high glucose medium (HyClone, United States). To complete the construction of the renal interstitial fibrosis model, NRK-52E was cultured in DMEM high glucose medium in a 37°C, 5% CO_2_, and 1% O_2_ hypoxia incubator.

The modeling time was 12 h, and the high, middle and low doses of TLYS were 500 μg/ml, 200 μg/ml, and 100 μg/ml, respectively, according to the CCK8 results. The cells were divided into six groups at random: 1) Control group: complete medium containing 10% fetal bovine serum in a normal incubator; 2) Model group: DMEM high glucose medium in a hypoxia incubator; 3) High dose group: DMEM high glucose medium +500 μg/ml of TLYS in a hypoxia incubator; 4) Medium dose group: DMEM high glucose medium +200 μg/ml of TLYS in a hypoxia incubator; 5) Low dose group: DMEM high glucose medium +100 μg/ml of TLYS in a hypoxia incubator; 6) MCC950 group: DMEM high glucose medium in a 10 μmol/L MCC950 in a hypoxia incubator.

### Western Blot Analysis

For western blot assays, renal tissues were lysed and homogenized in RIPA buffer supplemented with protease inhibitor cocktail l (C0001-1, Targetmol, China) and quantified with a BCA kit (P0013C, Beyotime, China). Protein sample extracts (30 mg/lane) were separated by SDS-PAGE and transferred onto a polyvinylidene difluoride membrane (PVDF). After the membranes were blocked with 5% BSA, they were incubated with the primary antibodies at 4°C overnight, followed by an HRP-conjugated secondary antibody. Then, the membranes were incubated with HRP-conjugated secondary antibody (Boster Biological Technology Co., Ltd., China) at room temperature for 1 h. Films were scanned by a ChemiScope 6,000 system (Qinxiang, Shanghai, China). ImageJ software was used to measure the protein bands based on that of GAPDH.

### Immunofluorescence Staining

Kidney sections were blocked with 10% goat serum at room temperature for 30 min, and then incubated with primary antibodies: GSDMD (1:200, ab255603, Abcam, United States), and IL-18 (1:200, 60070-1-Ig, Proteintech, United States) overnight at 4°C. Later on, the slides were incubated with corresponding secondary antibodies for 30 min. Finally, the slides were stained with DAPI solution for 10 min and captured by a laser scanning confocal fluorescence microscope (Olympus FV 1000, Japan).

### Statistical Analysis

For statistical analysis, the GraphPad Prism software was used. The mean standard deviation (SD) is used to express quantitative data. For all experimental data, one-way ANOVA was used, followed by Dunnett’s test. A *p* value of less than 0.05 was deemed significant.

## Results

### TLYS Alleviated Renal Function, Renal Injury, and Fibrosis in the UUO Rats

**FIGURE 1:**
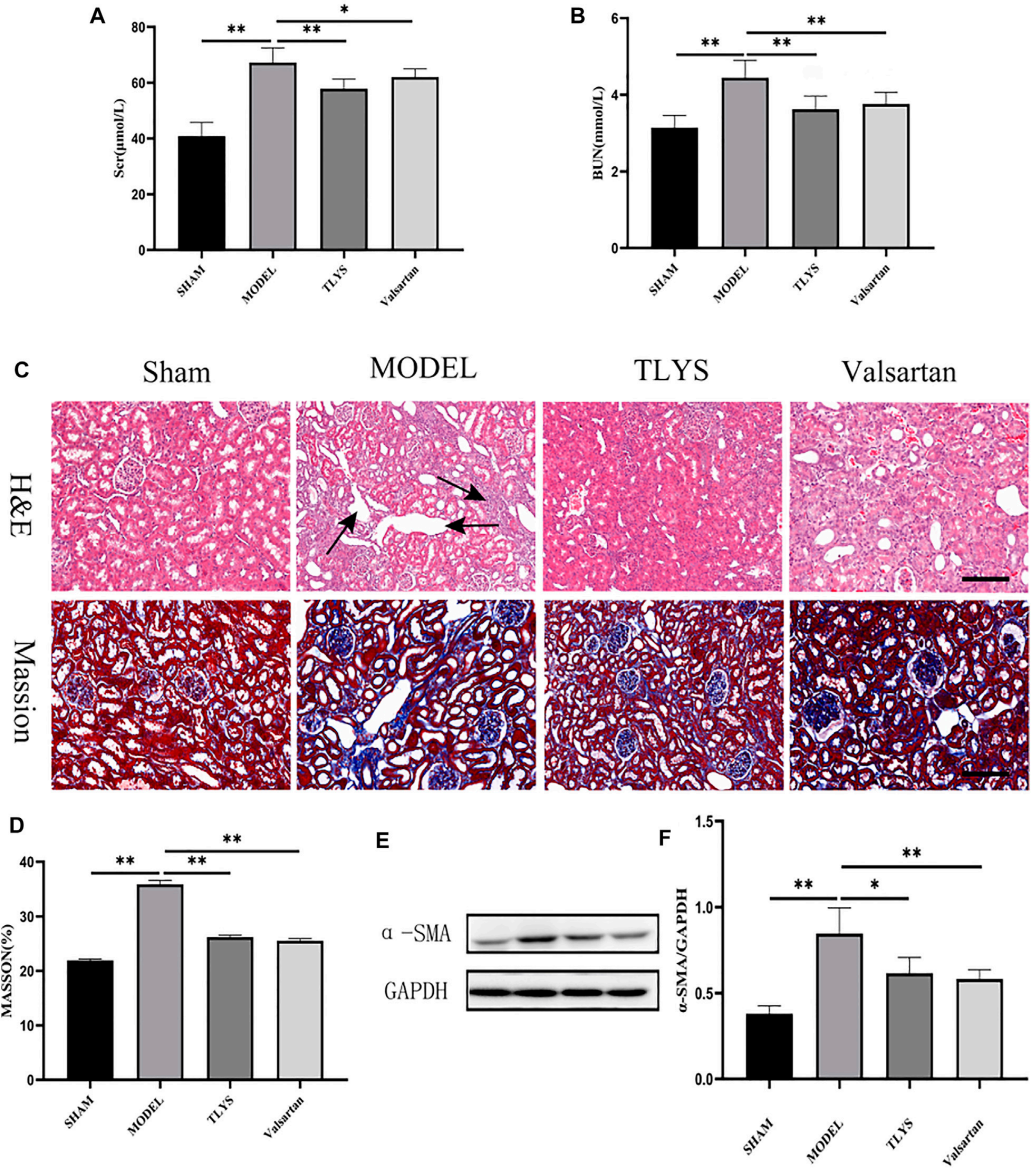
TLYS decoction alleviates the renal function, pathological kidney injuries and renal fibrosis in UUO rats. **(A,B)** serum creatinine and BUN were detected (n = 8). **(C)** H&E and Masson’s trichrome were performed to describe and evaluate kidney injury. **(D)** Collagen areas were quantified by Masson’s trichrome staining (n = 6). **(E,F)** The protein levels of α-SMA were assayed by Western blot and analyzed semi-quantitatively (n = 6). The magnification of the images is ×200, scale bar = 50 μm. Data were presented as means ± SD. *p < 0.05, **p < 0.01.

In comparison to the sham group, serum creatinine and urea levels were considerably higher in UUO rats, and renal function was dramatically improved after TLYS intervention. (Figures 1A,B). When compared to the sham group, HE and Masson’s trichrome staining revealed inflammatory cell infiltration and interstitial fibrosis in the kidney tissue of UUO rats, while TLYS intervention markedly alleviated interstitial fibrosis (Figures 1C,D). α-SMA is a protein that is commonly found in activated fibroblasts. Figures 1E,F shows that α-SMA protein expression was significantly upregulated in kidney tissues of UUO rats and that TLYS intervention improved its abnormal expression. These findings show that TLYS has a more beneficial effect on the kidneys of UUO rats.

### TLYS Attenuated Hypoxic NRK-52E Cells Injury and Fibrosis

**FIGURE 2:**
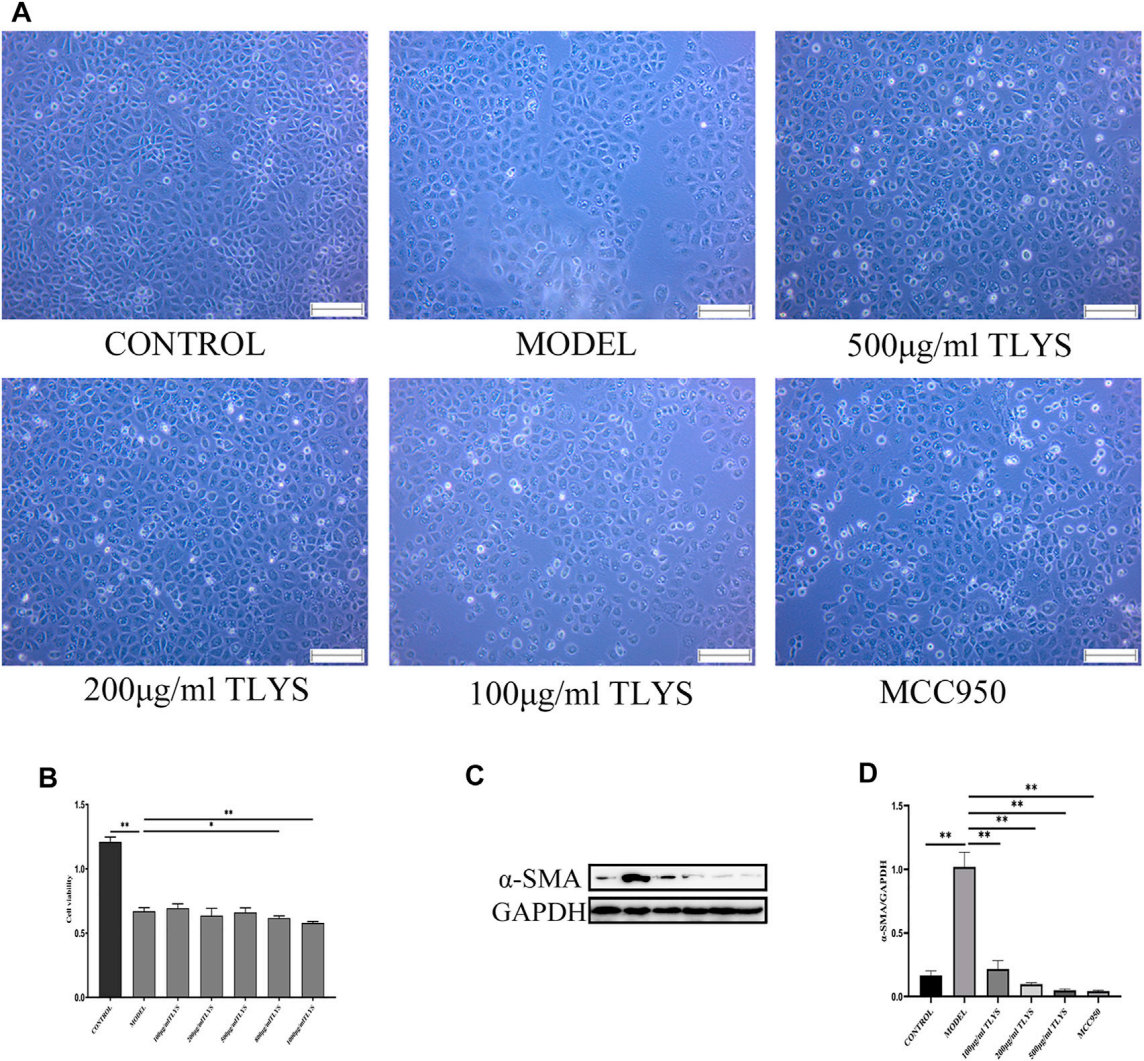
TLYS decoction affects the morphology, viability and fibrosis of NRK-52E cells induced by hypoxia. **(A)** The morphology of NRK-52E cells induced by hypoxia were observed by microscope. **(B)** The viability of NRK-52E cells induced by hypoxia were affected by cck8 test. **(C,D)** The protein levels of α-SMA were assayed by Western blot and analyzed semi-quantitatively (n = 3). The magnification of the images is ×100, scale bar = 50 μm. Data were presented as means ± SD. *p < 0.05, **p < 0.01.

We further investigated whether TLYS ameliorates hypoxia-induced renal epithelial cell injury. A cell viability experiment demonstrated that TLYS concentrations of 0–500 μg/ml had minimal influence on cell viability (Figure 2B). Cell viability was lower in hypoxic-treated NRK-52E cells than in normoxic NRK-52E cells, whereas cell viability was significantly lower in normoxic NRK-52E cells after treatment with 800 and 1000 μg/ml TLYS (Figure 2B). These findings suggested that excessive levels of TLYS are harmful to cells. Up to a dosage of 500 μg/ml, treatment with TLYS maintained cell viability in hypoxic NRK-52E cells (Figure 2B), whereas greater concentrations of TLYS decreased cell viability. As a result, 500, 200, and 100 μg/ml were chosen as the high, medium, and low concentration groups, respectively. Necrotic cells appeared to be on the rise in the model group, with rounded cell shape and the production of granular material and vacuoles in control cells. TLYS in various doses and MCC950 improved cellular morphology (Figure 2A). Western blot was used to assess ECM accumulation (Figure 2C). α-SMA was significantly higher in the model group compared to the control group but were reduced by TLYS treatment (Figures 2C,D).

### TLYS Reverses the Increased Pyroptosis in UUO Rat Renal Injury

**FIGURE 3:**
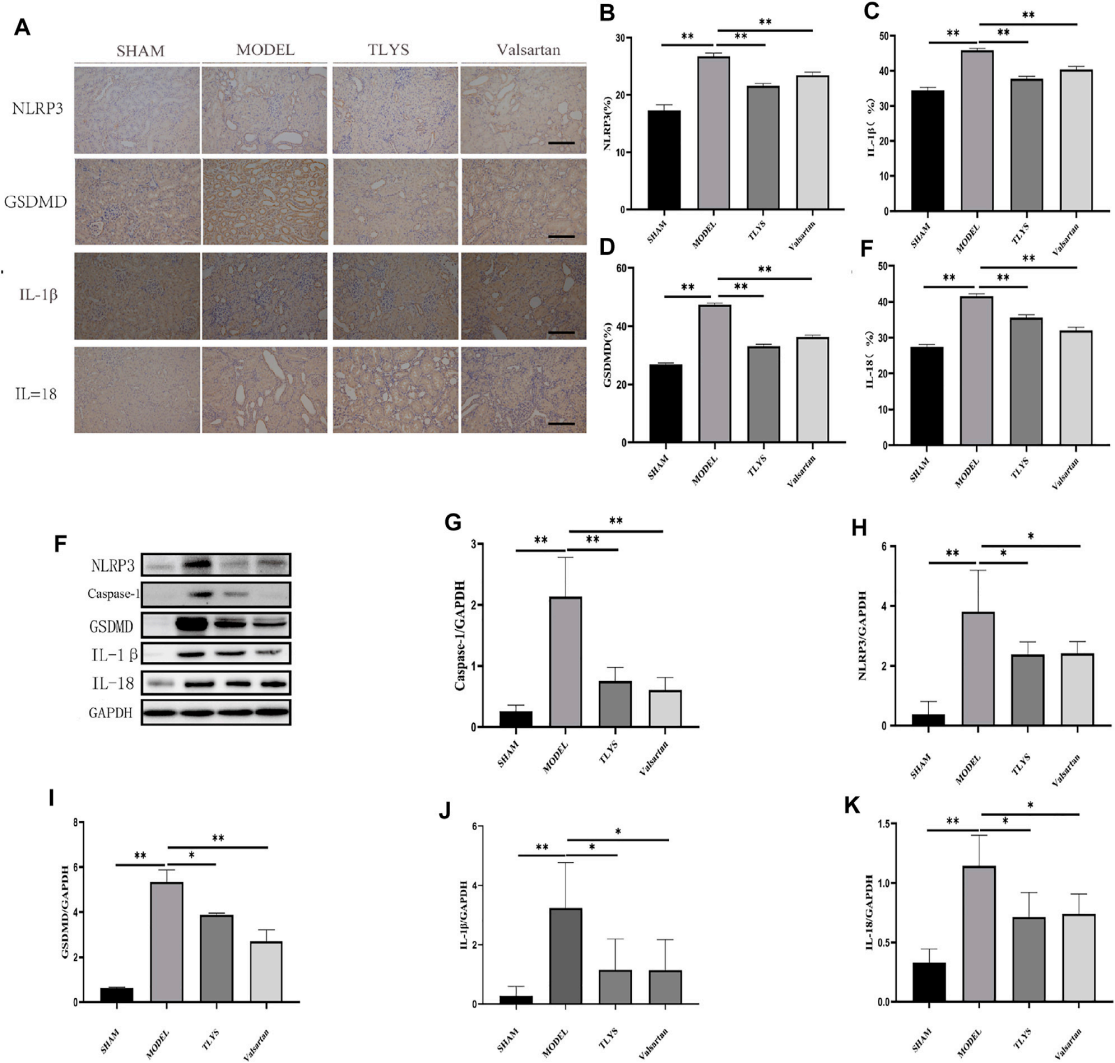
TLYS decoction suppresses the pyroptosis associated protein expressions in UUO rats. **(A–E)** Expression levels of NLRP3, GSDMD, IL-18 and IL-1β in the kidney were detected by immunohistochemistry and the optical intensity of the abovementioned proteins was measured (n = 6). **(F–K)** The protein levels of NLRP3, caspase-1, GSDMD, IL-18 and IL-1β in the kidney were assayed by Western blot and analyzed semi-quantitatively (n = 3). The magnification of the images is ×200, scale bar = 50 μm. Data were presented as means ± SD. *p < 0.05, **p < 0.01.

Numerous studies have demonstrated that NLRP3-mediated pyroptosis plays a role in the renal inflammatory response and renal injury. Immunohistochemistry and western blot were utilized to further analyze the expression of pyroptosis marker proteins to see if TLYS ameliorates renal interstitial fibrosis by modulating the pyroptosis pathway in renal cells. Immunohistochemistry showed that NLRP3, GSDMD, and the pro-inflammatory cytokines IL-1β and IL-18 protein expression were significantly higher in the model group compared to the sham group; Compared to the model group, GSDMD, IL-1β, and IL-18 protein expression were significantly lower in the TLYS and Valsartan groups (Figures 3A–E). We then used a Western blot assay to look at the expression levels of NLRP3, GSDMD, IL-1β, IL-18, and caspase-1 proteins, and the results were consistent with the immune tissues, as the levels of NLRP3, GSDMD, IL-1β, IL-18 and caspase-1 proteins in the UUO group were significantly higher than those in the Sham group, while those in the TLYS group were significantly lower than those in the UUO group (Figures 3F–K). Similarly, valsartan treatment significantly reduced the level of NLRP3, GSDMD, IL-1, IL-18, and caspase-1 proteins (Figures 3F–K).

### The Effects of TLYS on Pyroptosis in Hypoxic NRK-52E Cells

**FIGURE 4:**
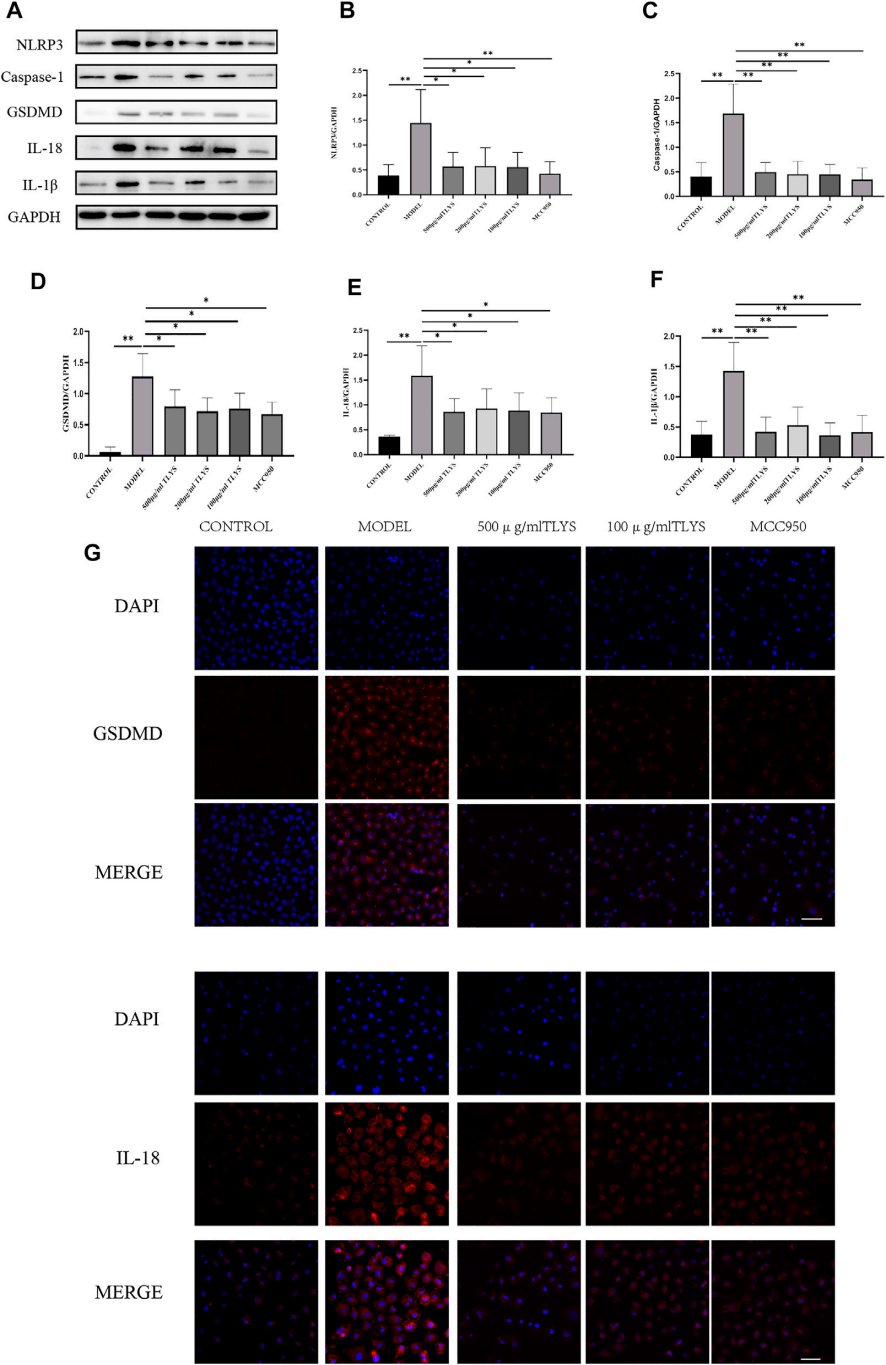
TLYS decoction suppresses the pyroptosis associated protein expressions of NRK-52E cells induced by hypoxia. **(A–F)** The protein levels of NLRP3, caspase-1, GSDMD, IL-18 and IL-1β in NRK-52E cells induced by hypoxia were assayed by Western blot and analyzed semi-quantitatively (n = 3). **(G)** The positions of GSDMD and IL-18 was labeled by immunofluorescence (n = 6). The magnification of the images is ×400, scale bar = 50 μm. Data were presented as means ± SD. *p < 0.05, **p < 0.01.

To evaluate the effect of TLYS on pyroptosis, we measured the expression of proteins related to pyroptosis activation, including NLRP3, GSDMD, IL-1β, IL-18, and caspase-1 proteins. The protein expression levels of NLRP3, GSDMD, IL-1β, IL-18, and caspase-1 significantly increased in hypoxic NRK-52E cells as compared to those in normoxic NRK-52E cells (Figures 4A–F). These decreases were ameliorated by treatment with TLYS at the dose of 500, 200, and 100 μg/ml (Figures 4A–F). GSDMD was found primarily in the cell membrane and cytoplasm, while IL-18 was found primarily in the cell plasma, according to immunofluorescence detection results (Figure 4G). GSDMD and IL-18 proteins were widely present in hypoxic NRK-52E cells, and their expression was significantly higher in the model group compared to the normal group; however, The expression was significantly lower in the TLYS (500 and 100 μg/ml) and MCC950 group compared to the model group (Figure 4G).

## Discussion

Inflammation has been proven in numerous studies to be important factor contributing to the progression of interstitial fibrosis in the kidney (Liu et al., 2018; Lv et al., 2018; Tang et al., 2019). Following tissue injury, infiltrating inflammatory cells are activated, resulting in the production of tissue-damaging molecules, such as reactive oxygen species (ROS) while inducing the production of fibrogenic cytokines and growth factors (Nathan and Ding, 2010; Schroder and Tschopp, 2010). The onset of pyroptosis in the UUO model may be caused by oxidative stress and inflammation (Chung et al., 2012; Zhang Y. et al., 2021). Recent studies have shown that renal inflammation can activate NLRP3 inflammatory vesicles (Kim et al., 2019; Tajima et al., 2019), which trigger innate immune defenses via pro-inflammatory cytokines such as IL-1β in response to signals such as infection and metabolic dysregulation (Vilaysane et al., 2010).

Our previous research has proved that TLYS has a good therapeutic effect on RIF (Jia et al., 2021). The present experimental study also found that TLYS could protect the kidney function and improve the histopathological damage of the kidney in UUO rats. Following UUO, NLRP3 knockout mice showed reductions in tubular apoptosis, inflammation, and fibrosis (Guo et al., 2017; Kim et al., 2018). At 14 days after UUO, leukocyte recruitment to the kidney was reduced, and inflammatory cytokines were reduced (Kim et al., 2018). The findings of this study also revealed that the expression of NLRP3 and caspase-1 was increased in the kidney tissues of rats in the model group, as was the content of inflammatory factors IL-1β and IL-18. TLYS treatment reduced the expression of NLRP3 and caspase-1 in rat kidney tissues, as well as the expression of inflammatory factors, and the interstitial fibrosis of the kidney. Meanwhile, A cell model was established in this study using hypoxia-induced RTEC injury for 12 h. Hypoxia caused cell morphology changes, structural damage, and decreased cell viability in RTEC; however, cell morphology changes and structural damage were improved in different TLYS concentration groups, indicating that TLYS could inhibit hypoxia-induced cell damage. The expression of IL-1β and IL-18 in the model group was significantly higher than in the control group, indicating that inflammation occurred in the model group. It has been reported that NLRP3, GSDMD, and caspase-1 was upregulated in RTEC stimulated by high glucose levels (Xie et al., 2019). We investigated the related proteins to see if hypoxia can cause inflammation by activating the NLRP3 pathway. The model group had higher levels of NLRP3, GSDMD, and caspase-1 expression than the control group, indicating that hypoxia can cause inflammation by activating the NLRP3 pathway. After 12 h of TLYS intervention at various concentrations, NLRP3, GSDMD, IL-1β, IL-18 and caspase-1 proteins were significantly reduced. It suggests that TLYS may inhibit NLRP3 and thus prevent the activation of pro-Caspase-1 to Caspase-1, resulting in the inability of IL-1β and IL-18 precursors to shear into active IL-1β and IL18. The present study found that pyroptosis occurred clearly in hypoxia-induced RTEC, and TLYS may exert its protective effect on hypoxia-induced RTEC by inhibiting the NLRP3-mediated pyroptosis pathway.

Current studies have shown that MCC950, a potent and selective small-molecule inhibitor of NLRP3, has therapeutic effects in several renal diseases, diabetes, and its complications. NLRP3 inflammasome is activated in podocytes of patients with lupus nephritis and mice, and inhibition of NLRP3 by MCC950 attenuates proteinuria, renal pathological damage, and podocyte fusion in mice with lupus nephritis (Fu et al., 2017). MCC950 decreased blood pressure and improved renal inflammation, renal fibrosis, and renal dysfunction in mice with hypertension model (Krishnan et al., 2016; Krishnan et al., 2019). MCC950 was used as a positive control drug in the current study to intervene in hypoxia-induced the tubular epithelial cells injury, and the results showed that cell fibrosis degree, as well as IL-1β and IL-18 levels, decreased in the inhibitor group compared to the model group. MCC950 may influence the development of tubular epithelial cell fibrosis by inhibiting the activation of NLRP3 inflammatory vesicles. MCC950 inhibits hypoxia-induced inflammatory response and fibrosis in RTECs by decreasing NLRP3 expression, Caspase-1 activation, inhibiting inflammatory mediators IL-1β and IL-18 expression, and eventually decreasing α-SMA expression.

Our team conducted the TLYS decoction fingerprint study by UHPLC-MS. A total of 37 compounds were identified (Jia et al., 2021). Studies have also shown that Salvianolic acid B can alleviate I/R injury in mice by inhibiting caspase-1/GSDMD-mediated pyroptosis via the Nrf2/NLRP3 signaling pathway (Pang et al., 2020). Endotoxemia-induced mortality and cardiomyopathy are reduced by Sodium tanshinone IIA sulfonate, which may be linked to NLRP3 inflammasome suppression (Chen et al., 2021). The protective mechanism of Hydroxysafflor yellow A in H/R-induced cardiomyocyte damage is linked to the NLRP3 inflammasome activation (Ye JX. et al., 2020). In Myocardial ischemia/reperfusion injury, Hydroxysafflor yellow A can activate AMPK to reduce the NLRP3 inflammasome via blocking the mTOR pathway (Ye J. et al., 2020). These ingredients may be the basis of TLYS inhibitors of pyroptosis. Herbal compounding, on the other hand, is the combination of several herbs with complex chemical compositions. These chemical components both promote and enhance the action of the drug, while also inhibiting and affecting the overall action of the drug. The glycosides extracted from the original solution of TLYS are primarily Salvianolic acid B, Sodium tanshinone IIA sulfonate, and Hydroxysafflor yellow A, but they may also contain other components. When the drug dosage is increased, these components may inhibit the anti-pyroptosis effect. However, the precise cause must be determined.

In conclusion, this study found that pyroptosis contributes to kidney damage following UUO. Furthermore, the findings demonstrated that TLYS exert significant effects on such injury via a mechanism closely related to the inhibition of the activation of the classical pyroptosis pathway mediated by the NLRP3 inflammasome.

## Data Availability

The original contributions presented in the study are included in the article/Supplementary Material, further inquiries can be directed to the corresponding authors.
